# Soy Protein Outperforms Whey Protein in Ameliorating Insulin Resistance but Not Obesity in High-Fat Diet-Induced Obese Mice

**DOI:** 10.3390/nu17213427

**Published:** 2025-10-31

**Authors:** Andong Ji, Yuxia Qi, Kuan Zhao, Juanjuan Niu, Runjia Shi, Zhongshi Qi, Liying Zhou, Chunhui Zhao, Duo Li

**Affiliations:** 1 Qingdao Public Health Clinical Center, Qingdao University, Qingdao 266033, China; jiandongqd@163.com (A.J.); 17669680091@163.com (Y.Q.); zhaokuan97@163.com (K.Z.); qingdaonjj@163.com (J.N.); 2 Institute of Nutrition and Health, Qingdao University, Qingdao 266071, China; srjcharacter@163.com (R.S.); qizhongshi0414@163.com (Z.Q.); zhouly90722@163.com (L.Z.); zch174199@163.com (C.Z.); 3 School of Public Health, Qingdao University, Qingdao 266071, China; 4 Tangshan Center for Disease Control and Prevention, Tangshan 063000, China; 5 Department of Food Science and Nutrition, Zhejiang University, Hangzhou 310058, China

**Keywords:** whey protein, soy protein, obesity, insulin resistance, therapeutic effect

## Abstract

**Background/Objectives:** To date, few studies have investigated the therapeutic effects of soy versus whey protein supplementation on obesity and insulin resistance (IR), yielding inconsistent findings. The aim of the present study was to compare the therapeutic efficacy of soy versus whey protein on obesity and IR and to elucidate their potential molecular mechanisms. **Methods:** Forty male C57BL/6J mice were randomly divided into two groups and fed either a normal diet (*n* = 8) or a high-fat diet (HFD, *n* = 32) for 16 weeks to induce obesity. After 16 weeks, HFD-induced obese mice were further randomized into three groups: HFD control, HFD + 20% whey protein isolate (WPI), and HFD + 20% soy protein isolate (SPI) for 6 weeks (*n* = 8). **Results:** Body weight, weight gain, body mass index, and Lee index showed no significant differences between the WPI and SPI groups. Compared with the WPI group, serum concentrations of insulin and leptin and the homeostasis model assessment of IR (HOMA-IR) were significantly lower, and thymus wet weight, fetal total cholesterol level, and serum glucose-dependent insulinotropic polypeptide concentration were significantly higher in the SPI group. Compared with the WPI group, the protein levels of GLUT4 and p-PI3K/PI3K were significantly higher in the SPI group. Metabolomics analysis showed that hepatic phosphocholine levels were significantly higher in the SPI group than in the WPI group. Moreover, hepatic differentially abundant metabolites of SPI- and WPI-fed mice were primarily enriched in the glycerophospholipid metabolism pathway. **Conclusions:** Soy protein was more effective than whey protein in ameliorating IR in HFD-induced obese mice, probably by modulating the PI3K-GLUT4 pathway and glycerophospholipid metabolism. Moreover, soy protein and whey protein showed comparable anti-obesity efficacy.

## 1. Introduction

The global prevalence of obesity has been rising dramatically. By 2030, it is projected that nearly 3 billion adults worldwide will be affected by overweight or obesity, of whom 1.1 billion will meet the criteria for obesity (defined as a body mass index [BMI] ≥ 30 kg/m^2^) [1]. As one of the primary complications of obesity, diabetes mellitus (DM) is estimated to affect 589 million adults aged 20–79 years globally in 2025, and this number is predicted to increase to 853 million by 2050 [2]. These staggering figures underscore the urgent need to develop effective strategies for preventing and treating obesity and DM.

High-protein diets (HPDs) have gained popularity as an effective approach to reducing weight gain and improving glycemic regulation. However, growing evidence suggests that the efficacy of HPDs against obesity and in regulating blood glucose homeostasis is substantially influenced by protein sources [3,4,5,6]. Consequently, identifying optimal protein sources is paramount to maximize the efficacy of HPDs in counteracting obesity and regulating blood glucose homeostasis. Insulin resistance (IR) is recognized as a central pathogenic driver of type 2 DM (T2DM) and represents a common complication of obesity [7,8]. Notably, IR and obesity exhibit a bidirectional pathogenic relationship [9]. Therefore, studies examining the protein-mediated anti-obesity effect should concurrently assess the impact of such intervention on IR. Whey protein and soy protein represent two widely consumed high-quality protein sources. Extensive research has demonstrated that whey or soy protein supplementation exerts beneficial metabolic effects, including reduction in body weight, regulation of lipid metabolism, antihypertensive effects, and enhancement of glucose homeostasis [10,11,12,13]. To date, a limited number of studies have compared the effects of whey and soy protein on weight gain reduction and glucose homeostasis regulation, yet their findings remain inconsistent [14,15,16,17,18,19,20,21]. Accordingly, we previously conducted comparative studies investigating the preventive effects of whey protein versus soy protein on obesity and IR in high-fat diet (HFD)-fed mice. Our results demonstrated that whey protein was more effective than soy protein in preventing obesity, while soy protein was more effective against IR [22,23]. Notably, some studies suggest that the efficacy of HPDs in counteracting obesity and improving glucose homeostasis may be influenced by the host’s obesity status [24]. In light of this potential influence of baseline obesity status, whether the conclusions from our previous study—demonstrating the preventive effects of whey protein and soy protein on obesity and IR in non-obese models—can be extrapolated to their therapeutic efficacy for established obesity and IR requires further experimental validation. Only two studies to date have directly compared the therapeutic effects of whey protein versus soy protein on obesity, with inconsistent findings. In one study, Aoyama et al. observed that after administering soy protein isolate (SPI) and whey protein isolate (WPI) diets for two weeks to yellow KK obese mice, body fat content and plasma glucose level were significantly lower in the SPI diet than in the WPI diet [20]. Conversely, Cain et al. reported opposite results in obese Zucker rats fed SPI or whey protein for 17 weeks and found that body weight and fat mass were higher in the SPI group than in the whey protein group [21]. Moreover, to date, no studies have compared the therapeutic effects of whey and soy protein on established IR in animal models, whereas our previous work explored their preventive roles [23].

In this context, the present study aimed to investigate the therapeutic effects of whey protein versus soy protein on obesity and IR, while elucidating underlying molecular mechanisms in HFD-induced obese mice.

## 2. Materials and Methods

### 2.1. Materials

The SPI used in this study was supplied by Shandong Shenxian Yuhua Biological Protein Co., Ltd. (Liaocheng, China) and contained 91.2 g protein, 2.0 g carbohydrate, 0.8 g fat, and 1.1 g ash per 100 g, with no detectable isoflavones. The WPI used in this study was supplied by MyProtein company (Manchester, UK) and contained 90.0 g protein, 2.5 g carbohydrate, 0.3 g fat, and 0.5 g ash per 100 g. The experimental mouse diets were supplied by Jiangsu Xietong Pharmaceutical Bio-engineering Co., Ltd. (Nanjing, China). Antibodies against AMP-activated protein kinase alpha (AMPKα) (#5831), p-AMPKα (#2535), phospho-ribosomal protein S6 kinase beta-1 (Thr389) (p-S6K1^Thr389^) (#9234), protein kinase B (AKT) (#4691), p-AKT^Ser473^ (#4060), phosphatidylinositol 3-kinase (PI3K) (#4257), p-PI3K (#4228), the insulin receptor substrate-1 (IRS-1) (#3407), and p-IRS-1^Ser307^ (#2381) were obtained from Cell Signaling Technology (Danvers, MA, USA). Antibodies against S6K1 (ab32529), the mammalian target of rapamycin (mTOR) (ab134903), and p-mTOR^Ser2448^ (ab109268) were obtained from Abcam (Cambridge, UK). Antibody against glucose transporter 4 (GLUT4) (YT5523) was obtained from Immunoway (Plano, TX, USA). Antibody against β-actin (AF7018) was obtained from Affinity (Nanjing, China). An HRP-conjugated goat anti-rabbit secondary antibody (abs20040) was obtained from Absin (Shanghai, China).

### 2.2. Animals and Diets

**Figure 1:**
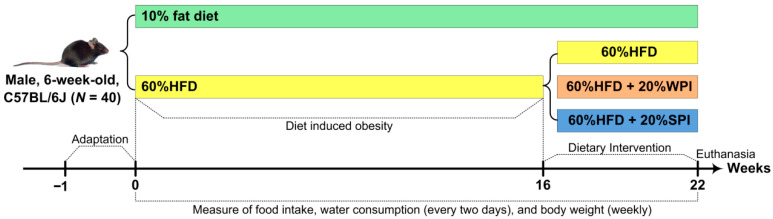
Flowchart of the present study design. SPI, soy protein isolate; WPI, whey protein isolate; HFD, high-fat diet.

Forty male C57BL/6J mice (6 weeks old) were sourced from Beijing Charles River Laboratory Animal Technology Co., Ltd. (Beijing, China) (animal license number: SCXK (Jing) 2021-0006) and acclimatized for 7 days. Animals were group-housed (4/cage) in controlled environments (21–23 °C, 45–55% humidity, 12 h light/dark cycle) with ad libitum access to standard chow and water. Post-acclimation, mice were randomized into two dietary groups: normal control group (*n* = 8) and HFD group (*n* = 32). The initial sample size of 8 mice per group for the final experimental groups was determined based on conventional practices in dietary protein intervention and metabolism studies [17,25,26]. Mice in the normal control group were fed a 10% fat diet (D12450J). Mice in the HFD group were fed a 60% fat diet (D12492). After 16 weeks of HFD feeding, obese mice (with body weight exceeding the average body weight of mice in the normal control group by 20%) [27] were selected and randomly divided into three groups (*n* = 8): HFD control, HFD + 20% WPI, and HFD + 20% SPI. Mice in the HFD + 20% WPI and HFD + 20% SPI groups received a 60% kcal fat diet, where casein was substituted by WPI or SPI, respectively. All HFDs were isocaloric. The experimental diet formulations are detailed in Supplementary Table S1. After 6 weeks of dietary intervention, all mice underwent a 12 h fast and were anesthetized via intraperitoneal injection of 3% (*w*/*v*) sodium pentobarbital. Blood was collected through enucleating the eyeball, after which euthanasia was performed by cervical dislocation. The body length was measured from the nasal tip to the anus. Subsequently, brown adipose tissue (BAT), inguinal white adipose tissue (iWAT), epididymal WAT (eWAT), mesenteric WAT (mWAT), perirenal WAT (pWAT), liver, stomach, thymus, spleen, and intestine were excised and weighed. All collected samples were immediately flash-frozen in liquid nitrogen and subsequently stored in a super-cold refrigerator until further use. The experimental flowchart is described in Figure 1. This experimental protocol was approved by the Animal Ethics Committee of Qingdao University (Approval No. 20230414C5714820230924015) and conducted in accordance with the Guidelines for Care and Use of Laboratory Animals of Qingdao University.

### 2.3. Food Intake, Water Consumption and Body Weight

Food intake and water consumption of mice were measured and recorded at the same time every two days. Mouse body weight was measured and recorded every Thursday morning. BMI, Lee index, and feed efficiency were calculated based on methods described in a previous study [22]. The relative weight of adipose tissue and organs was calculated using the following formula: Relative weight (%) = [Tissue/organ weight (g)/body weight (g)] × 100.

### 2.4. Serum Parameter Analysis

A HGM-114 blood glucose meter (Omron, Suzhou, China) was used to measure fasting blood glucose. Serum concentrations of triglyceride (TG), total cholesterol (TC), high-density lipoprotein cholesterol (HDL-C), low-density lipoprotein cholesterol (LDL-C), creatinine (CRE), blood urea nitrogen (BUN), aspartate aminotransferase (AST), and alanine aminotransferase (ALT) were determined using commercial assay kits (Nanjing Jiancheng Bioengineering Institute, Nanjing, China) following the manufacturer’s protocols. Serum concentrations of insulin, interleukin-1 beta (IL-1β), interleukin-6 (IL-6), interleukin-10 (IL-10), tumor necrosis factor alpha (TNF-α), leptin, adiponectin, cholecystokinin (CCK), glucagon-like peptide 1 (GLP-1), glucose-dependent insulinotropic polypeptide (GIP), and ghrelin were determined using commercial enzyme-linked immunosorbent assay (ELISA) kits (Jiangsu Jingmei Biotechnology Co., Ltd., Yancheng, China). The homeostasis model assessment of IR (HOMA-IR) was calculated using the following formula: HOMA-IR = fasting blood glucose (mmol L^−1^) × fasting insulin (µIU mL^−1^)/22.5 [22,28].

### 2.5. Histopathology

Fresh adipose and liver tissues were immersion-fixed in 4% paraformaldehyde at room temperature overnight, embedded in paraffin, and sectioned at 6 µm. Subsequently, deparaffinized sections were stained with hematoxylin and eosin (H&E) and Oil Red O. For each tissue section, five non-overlapping fields were randomly selected and imaged using an Olympus BX53F microscope (Tokyo, Japan). Then, a pathologist blinded to sample allocation assessed the images from each section. Histopathological changes in adipose and liver tissues were subsequently analyzed and quantified using ImageJ (Version 154-win-java8) and Image-Pro Plus 6.0 software.

### 2.6. TG and TC Levels in Feces and Liver Tissues

Fecal samples were collected from each mouse on the final day of the dietary intervention period. TG and TC levels in liver tissues and fecal samples were enzymatically quantified using commercial assay kits (Nanjing Jiancheng Bioengineering Institute, Nanjing, China). Briefly, 50 mg samples were homogenized in 450 µL ice-cold physiological saline (0.9% NaCl), and centrifuged at 2500 rpm for 10 min at 4 °C. The supernatants were reacted with their respective TG or TC assay working solutions in 96-well plates. After incubation at 37 °C for 10 min, the absorbance was measured at 510 nm using a microplate reader (Tecan Spark, Salzburg, Austria). Hepatic lipid levels were normalized to protein concentrations in liver homogenates, while fecal lipid levels were normalized to fecal sample dry weight.

### 2.7. Western Blotting

Western blotting was performed as previously described [23]. Briefly, liver tissues were homogenized in RIPA lysis buffer (Beyotime, Shanghai, China) and incubated on ice for 30 min. After centrifugation at 14,000× *g* for 10 min (4 °C), supernatants were collected for protein quantification using a BCA assay kit (Epizyme, Shanghai, China). Protein lysates were mixed with 5× loading buffer (Solarbio, Beijing, China) at a 4:1 ratio, denatured at 100 °C for 7 min, and electrophoresed on 8–12% SDS-PAGE gels with equal protein loading (15–30 µg/lane). Proteins were transferred to PVDF membranes, blocked with 5% skim milk for 1 h at room temperature, and washed three times with TBST. Membranes were incubated with primary antibodies (overnight at 4 °C) followed by secondary antibodies (for 2 h at room temperature). Protein bands were detected using Fusion Solo S (Vilber Lourmat, Fontenay-sous-Bois, France) and quantified with ImageJ (NIH, Bethesda, MD, USA).

### 2.8. Metabolomics Analysis

Serum samples (20 μL) were mixed with 300 μL ice-cold acetonitrile and centrifuged at 12,000× *g* for 15 min at 4 °C. Liver tissues (100 mg) were homogenized in 500 μL ice-cold methanol/water (1:1, *v*/*v*) and centrifuged under identical conditions. Supernatants from both sample types were filtered through 0.22 μm syringe filters into amber vials. Quality control (QC) samples were prepared by pooling 10 μL aliquots of each filtered sample. Metabolomics profiling was performed using an Agilent Technologies 6530C Q-TOF LC/MS (Agilent Technologies, Santa Clara, CA, USA). Samples (3 μL) were separated on an ACQUITY UPLC BEH C18 Column (2.1 mm × 100 mm, 1.7 μm) (Waters, Milford, MA, USA) under a positive ESI detection mode ([M+H]^+^). The flow rate was set to 400 μL/min and the column temperature to 55 °C. The mobile phases consisted of: (A) water containing 0.1% formic acid and (B) acetonitrile containing 0.1% formic acid. The elution gradient was initially maintained at 5% B for 1 min, ramped linearly to 95% B over 15 min, held for 2 min, returned to 5% B at 17.1 min, and re-equilibrated for 5 min. In addition, the mass spectrometry parameters were configured as follows: nitrogen gas flow rate of 11 L/min, capillary voltage 4 kV, scan range m/z 50–1500 with an acquisition rate of 1 spectrum/s, scan time 0.2 s, resolution 100,000 (FMHW), capillary temperature 320 °C, and auxiliary gas heater temperature 350 °C. Samples were injected in random order, with QC samples inserted at intervals of every eight samples throughout the analytical run. Data acquisition was performed using Mass Hunter (Agilent Technologies, Santa Clara, CA, USA) and converted via AbfConverter software (Version 1.3.8550). Metabolite identification was performed based on metabolomics databases in mass spectrometry-data independent analysis software (MS-DIAL, Version 2.54). Processed data were further analyzed using MS-FLO (https://msflo.fiehnlab.ucdavis.edu) (accessed on 14 October 2025) and subsequently subjected to comprehensive analysis using MetaboAnalyst 6.0 (https://www.metaboanalyst.ca) (accessed on 14 October 2025). The workflow in MetaboAnalyst comprised: sample normalization, log-transformation, Pareto scaling, principal component analysis (PCA), and orthogonal partial least squares-discriminant analysis (OPLS-DA). Differentially abundant metabolites (DAMs) among experimental groups were screened using two criteria: (1) variable importance in projection (VIP) scores from the OPLS-DA model > 2, and (2) a between-group significance level of *p* < 0.05. To enhance the reliability of metabolite identification and address concerns regarding exogenous compounds, stricter filtering and validation procedures were incorporated into the metabolomic data processing workflow: after preprocessing the raw mass spectrometry data, only metabolite signals with a peak intensity > 3000 were retained to exclude low-confidence signals potentially derived from background noise. For metabolite identification, strict criteria were applied, including a mass spectrum match score > 95% (based on MS/MS fragment ion similarity) against endogenous metabolite databases such as HMDB, and a retention time deviation < 2 s relative to database references. Additionally, potential exogenous substances were systematically screened and excluded by cross-referencing with the DrugBank and PubChem database, ensuring that only authentic endogenous metabolites of mice were included in the final analysis. KEGG pathway enrichment analysis was subsequently performed on all identified DAMs.

### 2.9. Statistical Analysis

All statistical analyses were performed using SPSS 21.0 (IBM SPSS, Chicago, IL, USA). Data are expressed as mean ± standard error of the mean (SEM) when normally distributed; otherwise, they are presented as median (interquartile range). When data were normally distributed, independent-samples t tests were applied for comparisons between two groups. For comparisons among three or more groups with normally distributed data, one-way analysis of variance (ANOVA) was used, followed by least significant difference (LSD) post hoc tests for pairwise comparisons when significant main effects were observed. When data deviated from normality, non-parametric tests were employed for data analysis. Food intake, energy intake, water consumption, and body weight were analyzed using repeated-measures ANOVA. Pearson correlation was used to evaluate associations between DAMs and body weight or IR-related parameters when the data followed a normal distribution; otherwise, Spearman’s rank correlation analysis was applied. A two-tailed *p* value < 0.05 was considered statistically significant.

## 3. Results

### 3.1. Effect of WPI and SPI on Body Weight

**Figure 2:**
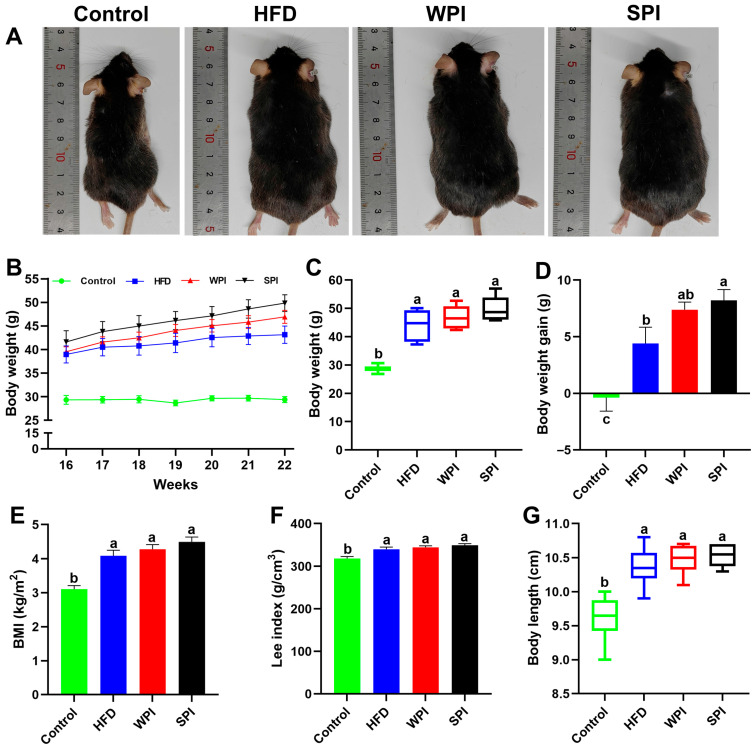
Effects of WPI and SPI on body weight-related parameters. (**A**) Representative pictures of mice from each group. (**B**) Body weight changes during the 6-week intervention. Body weight (**C**), body weight gain (**D**), BMI (**E**), the Lee index (**F**), and body length (**G**) of mice after 6-week intervention. BMI, body mass index. Data represent mean ± SEM or median (interquartile range) (*n* = 6–8). During the intervention period, two mice in the SPI group were excluded due to abnormal weight loss. Different lowercase letters indicate significant differences (*p* < 0.05).

After 6 weeks of intervention, no significant differences existed in body weight, weight gain, BMI, Lee index, or body length between the WPI and SPI groups. However, the SPI group exhibited significantly higher body weight gain than the HFD group (Figure 2).

### 3.2. Effect of WPI and SPI on Food Intake, Energy Intake and Water Consumption

**Figure 3:**
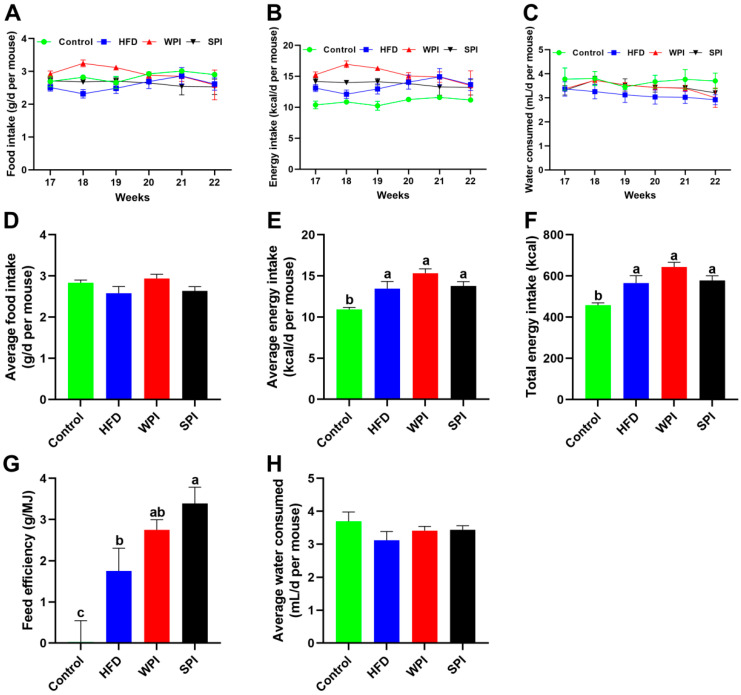
Effect of WPI and SPI on food intake, energy intake and water consumption. Dynamic changes in the average daily food intake (**A**), energy intake (**B**), and water consumption (**C**) during the 6-week intervention. Average daily food intake (**D**), average daily energy intake (**E**), total energy intake (**F**), feed efficiency (**G**), and average daily water consumption (**H**) throughout the intervention period. Data are represented as mean ± SEM (*n* = 6–8). Different lowercase letters indicate significant differences (*p* < 0.05).

No significant differences were observed in the average daily food intake, average daily energy intake, average daily water consumption, total energy intake, and feed efficiency between the WPI and SPI groups (Figure 3).

### 3.3. Effect of WPI and SPI on Adipose Tissue and Organ Mass

**Figure 4:**
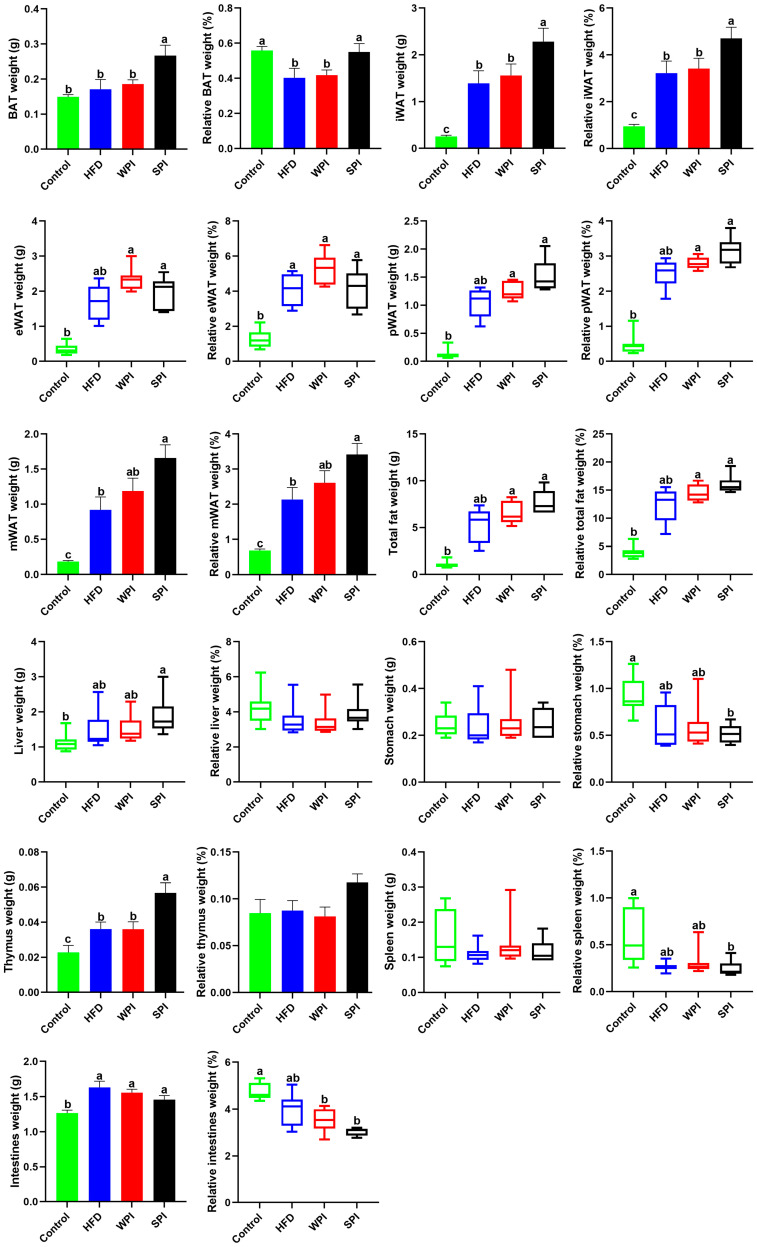
Effect of WPI and SPI on adipose tissue and organ mass. BAT, brown adipose tissue; iWAT, inguinal white adipose tissue; eWAT, epididymal white adipose tissue; pWAT, perirenal white adipose tissue; mWAT, mesenteric white adipose tissue. Data represent mean ± SEM or median (interquartile range) (*n* = 6–8). Different lowercase letters indicate significant differences (*p* < 0.05).

Compared with the SPI group, the wet weight and relative weight of BAT and iWAT, along with the wet weight of thymus, were significantly lower in the WPI group. No significant differences were observed in the relative weight of thymus, as well as in the wet weight and relative weight of eWAT, pWAT, mWAT, total fat, liver, stomach, spleen, and intestines between the WPI group and the SPI group (Figure 4).

### 3.4. Effect of WPI and SPI on Serum Parameters

**Figure 5:**
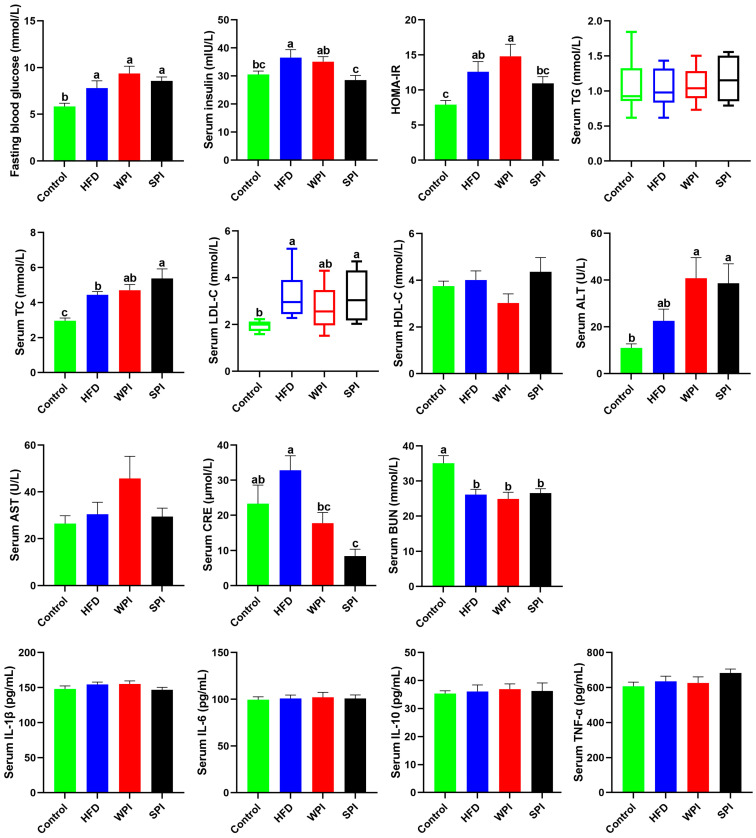
Effect of WPI and SPI on serum parameters. HOMA-IR, the homeostasis model assessment of insulin resistance; TG, triglyceride; TC, total cholesterol; LDL-C, low-density lipoprotein cholesterol; HDL-C, high-density lipoprotein cholesterol; ALT, alanine aminotransferase; AST, aspartate aminotransferase; CRE, creatinine; BUN, blood urea nitrogen; TNF-α, tumor necrosis factor alpha. Data represent mean ± SEM or median (interquartile range) (*n* = 6–8). Different lowercase letters indicate significant differences (*p* < 0.05).

Compared with the control group, the concentrations of fasting blood glucose and serum insulin, as well as the HOMA-IR level, were significantly higher in the HFD group. Compared with the WPI group, the SPI group exhibited significantly lower serum insulin concentration and lower HOMA-IR. Serum ALT level was significantly elevated in both the WPI and SPI groups relative to the control group, although no significant difference was observed between the two treatment groups. Serum CRE level was significantly lower in the SPI group compared to both the control and HFD groups. No significant differences existed in serum concentrations of TG, TC, LDL-C, HDL-C, AST, CRE, and BUN between the WPI and SPI groups. No significant differences were found in serum concentrations of IL-1β, IL-6, IL-10, and TNF-α among any of the groups (Figure 5).

### 3.5. Effect of WPI and SPI on Serum Appetite-Related Hormone Levels

**Figure 6:**
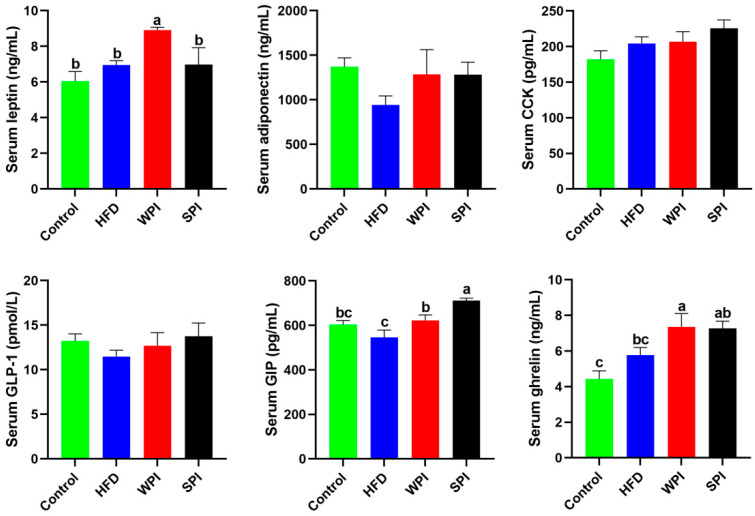
Effect of WPI and SPI on serum appetite-related hormone levels. CCK, cholecystokinin; GLP-1, glucagon-like peptide 1; GIP, glucose-dependent insulinotropic polypeptide. Data are represented as mean ± SEM (*n* = 6–8). Different lowercase letters indicate significant differences (*p* < 0.05).

Compared with the WPI group, the SPI group exhibited significantly lower serum leptin concentration, but significantly higher serum GIP concentration. No significant differences were observed in serum concentrations of adiponectin, CCK, GLP-1, and ghrelin between the WPI and SPI groups (Figure 6).

### 3.6. Effects of WPI and SPI on Fecal TG and TC Levels

**Figure 7:**
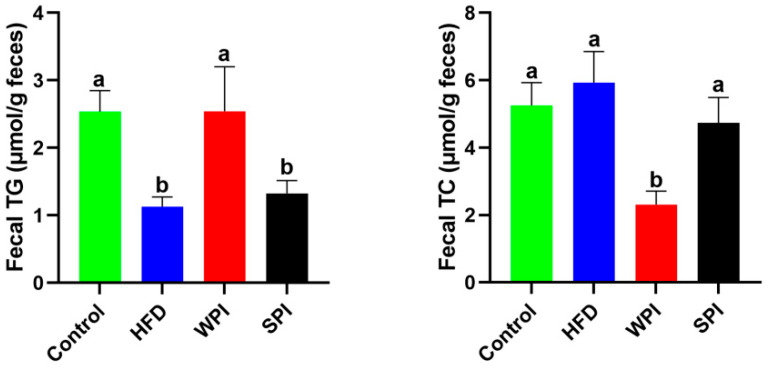
Effects of WPI and SPI on fecal TG and TC levels. TG, triglyceride; TC, total cholesterol. Data are represented as mean ± SEM (*n* = 6). Different lowercase letters indicate significant differences (*p* < 0.05).

Fecal TG levels in the SPI group were significantly lower than those in the WPI group, whereas TC levels were significantly higher (Figure 7).

### 3.7. Effects of WPI and SPI on Adipose Tissue Morphology

**Figure 8:**
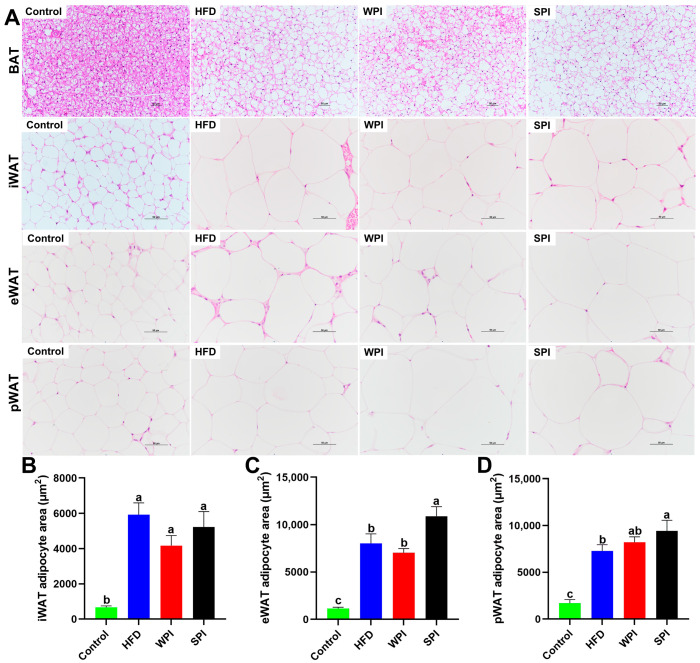
Effects of WPI and SPI on adipose tissue morphology. (**A**) Representative H&E-stained sections of BAT, iWAT, eWAT, and pWAT from each group after 6-week intervention. Adipocyte area in iWAT (**B**), eWAT (**C**), and pWAT (**D**). Scale bar: 50 μm. BAT, brown adipose tissue; iWAT, inguinal white adipose tissue; eWAT, epididymal white adipose tissue; pWAT, perirenal white adipose tissue. Data are represented as mean ± SEM (*n* = 3). Different lowercase letters indicate significant differences (*p* < 0.05).

Compared with the control group, the HFD, WPI, and SPI groups exhibited marked accumulation of lipid droplets in BAT and significant enlargement of adipocyte area in iWAT, eWAT, and pWAT. Furthermore, the SPI group exhibited increased lipid droplets in BAT and enlarged adipocyte area in eWAT relative to the WPI group (Figure 8).

### 3.8. Effects of WPI and SPI on Liver Morphology

**Figure 9:**
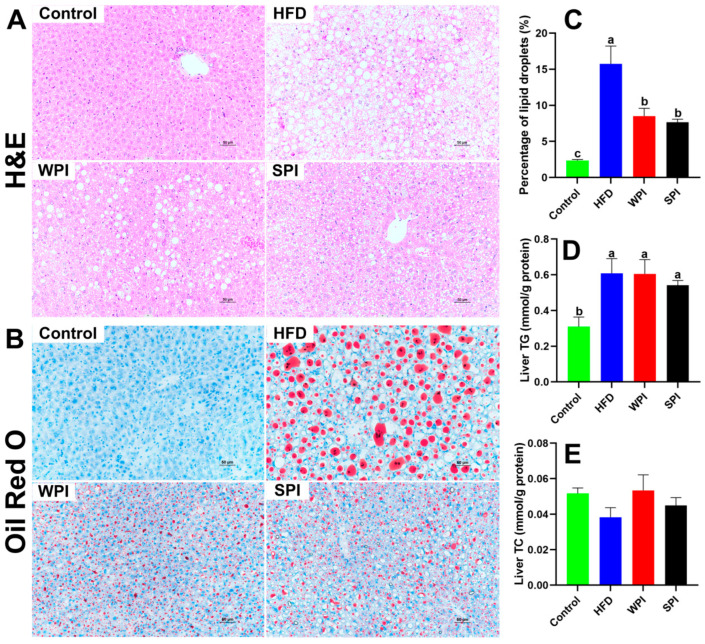
Effects of WPI and SPI on liver morphology. Representative H&E-stained (**A**) and Oil Red O-stained (**B**) sections of liver from each group after 6-week intervention. (**C**) Percentage of lipid droplets in the liver (*n* = 3). Scale bar: 50 μm. Hepatic TG (**D**) and TC (**E**) levels (*n* = 6). TG, triglyceride; TC, total cholesterol. Data are represented as mean ± SEM. Different lowercase letters indicate significant differences (*p* < 0.05).

H&E and Oil Red O staining revealed that HFD significantly induced hepatic steatosis. Compared with both the WPI and SPI groups, the HFD group demonstrated more severe hepatic pathology, characterized by increased hepatocyte swelling, increased cytoplasmic vacuolization, and increased lipid droplets accumulation. No significant differences were observed between the WPI and SPI groups in the percentage of lipid droplets in the liver or hepatic TG and TC levels (Figure 9).

### 3.9. Effects of WPI and SPI on the Expression of Hepatic Insulin Signaling-Related Proteins

**Figure 10:**
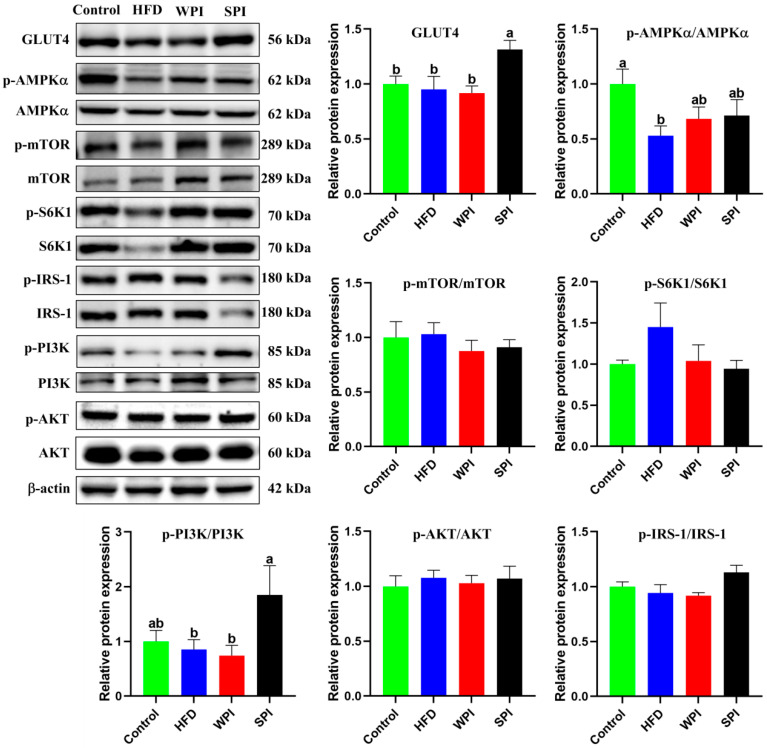
Effects of WPI and SPI on the expression of hepatic insulin signaling-related proteins. Protein expression was assessed by Western blotting. Data are represented as mean ± SEM (*n* = 6). Different lowercase letters indicate significant differences (*p* < 0.05).

The SPI group exhibited significantly higher expression levels of GLUT4 and the p-PI3K/PI3K ratio compared with the WPI group. There were no significant differences in the ratios of p-AMPKα/AMPKα, p-mTOR/mTOR, p-S6K1/S6K1, p-AKT/AKT, and p-IRS-1/IRS-1 between the WPI and SPI groups (Figure 10).

### 3.10. Effects of WPI and SPI on Serum and Liver Metabolic Profiles

**Figure 11:**
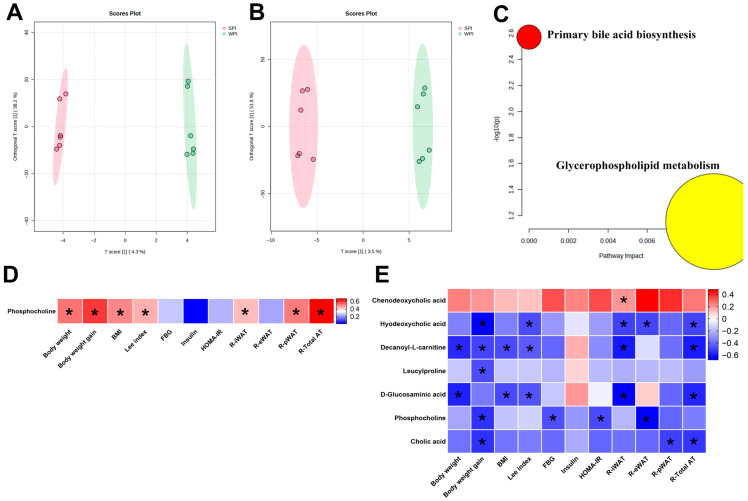
Effects of WPI and SPI on serum and liver metabolic profiles. Orthogonal partial least squares-discriminant analysis (OPLS-DA) of WPI vs. SPI groups in serum (**A**) and liver (**B**). (**C**) Disturbed metabolic pathways in the WPI vs. SPI groups in liver. Heatmap analysis of the Pearson correlation and Spearman’s rank correlation between differentially abundant metabolites (DAMs) and body weight or IR-related parameters in serum (**D**) and liver (**E**). BMI, body mass index; FBG, fasting blood glucose; HOMA-IR, the homeostasis model assessment of insulin resistance; R-iWAT, relative iWAT weight; R-eWAT, relative eWAT weight; R-pWAT, relative pWAT weight; R-Total AT, relative total fat weight. * *p* < 0.05 (*n* = 6 per group).

OPLS-DA score plots of serum and hepatic samples demonstrated distinct clustering separation between the WPI and SPI groups, indicating significant differences in their metabolic profiles (Figure 11A,B). However, the PCA score plot showed a high degree of overlap between the WPI and SPI groups, which confirms that there is no distinct global metabolic separation between the two groups (Supplementary Figure S1). Notably, permutation tests (*n* = 100) were performed to evaluate model quality, and the results indicated a risk of overfitting (serum: *R*^2^*Y* = 0.998, *Q*^2^ = −0.664; liver: *R*^2^*Y* = 0.994, *Q*^2^ = 0.286). In serum, the level of phosphocholine was significantly higher in the SPI group compared with the WPI group (Supplementary Table S2). In liver tissue, seven DAMs were identified. Phosphocholine exhibited significantly higher levels in the SPI group, while chenodeoxycholic acid, hyodeoxycholic acid, decanoyl-L-carnitine, leucylproline, D-glucosaminic acid, and cholic acid showed significantly lower levels relative to the WPI group (Supplementary Table S3). In addition, KEGG enrichment analysis revealed that DAMs in the livers of WPI- and SPI-fed mice were significantly enriched in the primary bile acid biosynthesis pathway and glycerophospholipid metabolism pathway (Figure 11C). Correlation analysis showed that serum phosphocholine levels were positively correlated with body weight-related parameters (Figure 11D). In the liver, chenodeoxycholic acid levels were positively correlated with R-iWAT; phosphocholine levels were negatively correlated with FBG and HOMA-IR; and hyodeoxycholic acid, decanoyl-L-carnitine, leucylproline, D-glucosaminic acid, phosphocholine, and cholic acid were significantly negatively correlated with body weight-related parameters (Figure 11E).

## 4. Discussion

In our previous study, the preventive effects of whey protein and soy protein against obesity and IR were compared at three dietary protein levels (10%, 20%, and 30% of total energy). It was found that the difference in their efficacy for obesity prevention was most pronounced at the 20% protein level. Furthermore, compared with the 10% and 30% protein levels, mice in the whey protein group fed a 20% protein diet exhibited lower serum insulin concentrations and HOMA-IR [22,23]. Based on these findings, the present study further investigated the therapeutic effects of whey protein and soy protein at the 20% protein level on obesity and IR in a mouse model. Our results demonstrated that while both protein sources exerted comparable anti-obesity effects, soy protein exhibited superior efficacy in ameliorating obesity-induced IR. This effect was potentially mediated through the PI3K-GLUT4 signaling pathway and glycerophospholipid metabolism. To our knowledge, this is the first study to compare the therapeutic effects of whey protein and soy protein on IR using an animal model—distinct from previous research that focused on their preventive effects against IR development [23].

The present study demonstrated that soy protein ameliorated obesity-induced IR more effectively than did whey protein, consistent with prior evidence [18,19]. Our prior studies demonstrated that under a “preventive intervention” paradigm (simultaneous HFD feeding and protein supplementation for 12 weeks), soy protein outperformed whey protein in preventing IR primarily by regulating lipid metabolism, the AMPK/mTOR pathway, and gut microbiota [23]. In contrast, the present study adopted a “therapeutic intervention” model (16 weeks of HFD-induced obesity/IR followed by 6 weeks of protein supplementation), which more closely mimics the clinical scenario of treating established metabolic disorders. This paradigm shift allowed us to explore the potential mechanisms by which soy protein ameliorates IR more deeply and directly. To elucidate the mechanisms underlying the superior efficacy of soy protein over whey protein in ameliorating IR under therapeutic intervention, we conducted further investigations. The thymus, a critical lymphoid organ, plays an indispensable role in modulating immune responses. Both thymic mass and the thymic index (thymus-to-bod y weight ratio) serve as direct morphological indicators of host immunocompetence, with higher values of these parameters typically associated with enhanced immune function [29,30,31]. Studies have established a close association between host immunocompetence and diabetic pathogenesis and shown that thymic index demonstrates a significant inverse correlation with HOMA-IR [32,33,34]. Notably, the impact of SPI on thymic function was unaddressed in our previous studies. In the present study, SPI-fed mice exhibited significantly higher thymus wet weight and a trend toward elevated thymic index compared with WPI-fed mice, suggesting enhanced immunocompetence. This novel finding implies a potential association between SPI-induced thymic enhancement and its anti-IR effects, highlighting immune regulation as a potential complementary pathway—particularly relevant in established obesity, a state of low-grade inflammation. However, the precise mechanisms by which thymic function modulates SPI-mediated IR improvement require further investigation. Chronic inflammation is closely associated with IR [35]. Therefore, we detected inflammation-related indicators and found that there were no significant differences in serum concentrations of IL-1β, IL-6, IL-10, and TNF-α between the SPI group and the WPI group. Several studies have reported a positive correlation between serum leptin levels and IR [36,37]. In the present study, serum leptin levels in the SPI group were significantly lower than those in the WPI group. This finding, consistent with previous research conclusions, partially explains why soy protein exhibits superior anti-IR efficacy compared to whey protein. In addition to leptin, the role of GIP, another factor associated with IR, also merits attention. Several studies have investigated the relationship between GIP levels and IR, yet the conclusions remain controversial [38,39,40]. In this study, the SPI group exhibited significantly higher serum GIP levels compared to the WPI group. However, the specific role of GIP in the anti-IR effect of soy protein requires further investigation. The IRS-1/PI3K/AKT pathway is a canonical insulin transduction pathway, with its dysregulation contributing to the development of IR [41,42]. AMPK activation enhances the IRS-1/PI3K/AKT signaling pathway, whereas mTOR activation suppresses it [43,44]. Our previous study demonstrated that soy protein ameliorates IR more effectively than whey protein by activating AMPK and inhibiting mTOR concomitantly, thereby restoring IRS-1/PI3K/AKT signaling [23]. To investigate whether soy protein exerts its IR-improving effect through the aforementioned mechanism in the present study, we quantified the expression of key signaling proteins. Our results revealed that the expression levels of GLUT4 and the p-PI3K/PI3K ratio were significantly higher in the SPI group compared with the WPI group. These data suggest that soy protein’s greater efficacy in ameliorating IR may involve activation of the PI3K-GLUT4 axis; these results align with our prior findings in highlighting the PI3K-GLUT4 axis as a critical mediator [23]. The absence of AMPK/mTOR/IRS-1/AKT activation (observed in preventive settings [23]) may stem from differences in intervention paradigms—specifically, a 12-week intervention period for prevention versus 6 weeks for treatment—as well as variations in the baseline metabolic status of the mice. Notably, the therapeutic intervention model displayed more severe baseline obesity and IR, with potential adaptive metabolic remodeling in the liver contributing to this discrepancy. Consistent with our findings, Das et al. demonstrated that the protein isolates from Hawaijar, a popular fermented soybean food of North-East India, exert significant antidiabetic effects by activating the PI3K/AKT/GLUT4 signaling pathway [45]. As is well known, the onset and progression of IR are closely linked to dysregulated metabolic activities. Metabolites—small-molecule organic compounds produced by a series of metabolic processes—provide dynamic snapshots of physiological and pathophysiological states [46]. Therefore, the detection of metabolites in systemic circulation or tissues holds promise for elucidating disease etiology and screening for associated biomarkers. To elucidate the potential mechanisms underlying the differential effects of whey protein and soy protein on IR from a metabolic perspective, we employed an untargeted metabolomics approach to analyze metabolite profiles in both serum and liver tissues of mice from the WPI and SPI groups. Intergroup comparisons and correlations between the identified DAMs and IR-related parameters (fasting blood glucose, insulin, or HOMA-IR) were then performed. Consequently, DAMs were identified in both serum and liver tissues. While serum DAMs showed no significant correlations with IR-related parameters, hepatic DAMs revealed specific associations: only phosphocholine exhibited significant negative correlations with IR-related parameters. Pathway analysis further revealed that hepatic DAMs were primarily enriched in the primary bile acid biosynthesis pathway and glycerophospholipid metabolism pathway. Notably, phosphocholine is a key metabolite within the glycerophospholipid metabolism pathway. A Chinese clinical study identified DAMs in plasma samples from pregnant women with gestational diabetes mellitus (GDM) compared to healthy controls. The results demonstrated that glycerophospholipids were the most prevalent class of dysregulated metabolites, with glycerophospholipid metabolism being a core metabolic pathway affected by GDM [47]. To date, several studies have also reported inverse associations between glycerophospholipids levels and blood glucose levels, as well as GDM risk and diabetes incidence [48]. These results further corroborate the findings of the present study. Taken together, these data suggest that soy protein ameliorates obesity-induced IR more effectively than whey protein, potentially through modulation of the glycerophospholipid metabolism pathway. However, a notable limitation of the initial metabolomic analysis in this study was the detection of several exogenous compounds, including 2-amino-4-nitrotoluene, orlistat, milrinone, ectoine, 10-methylacridone, lidocaine, and retapamulin, which have been confirmed through rigorous validation to be non-endogenous in mice. Through retrospective analysis of laboratory workflows, we speculate that potential sources of trace contamination—such as shared consumables and environmental residues (e.g., drug aerosols, volatile reagents in laboratory air) that may have adsorbed onto samples during processing—could account for the detection of these exogenous compounds. Importantly, these exogenous compounds have been excluded from the final set of identified DAMs, and their initial detection did not affect the characterization of endogenous metabolites or the interpretation of key metabolic pathways.

In addition, the present study revealed comparable efficacy between soy protein and whey protein in counteracting obesity. Similar effects of whey protein and soy protein on obesity treatment have also been reported by others. In an animal study, genetically obese mice were fed an energy-restricted low fat and high protein (35% WPI or SPI) diet for 2 weeks. No significant differences in final body weight or total weight gain were found between groups [20]. In a randomized clinical trial of 90 free-living overweight/obese adults, 23 weeks of soy protein versus whey protein supplementation resulted in no significant differences in body weight or fat mass [15]. Notably, some studies have also reported inconsistent results. In an animal study, obese Zucker rats fed SPI or whey protein for 17 weeks showed significantly higher body weight and total adiposity in the SPI group than in the whey protein group [21]. In a randomized controlled trial of 45 overweight and obese men, supplemental preloads of whey protein concentrate consumed 30 min prior to the ad libitum main meal were more beneficial than SPI preloads for reducing appetite, body weight, BMI, waist circumference, and body fat mass [14]. Differences in the sample size, the dosage and purity of proteins, the duration of interventions, the physiological status of subjects, and the use of animal models may contribute to the observed discrepancies in study results.

In the present study, although body weight gain did not differ significantly between the WPI group and the SPI group, this parameter was significantly elevated in the SPI group compared with the HFD group, whereas no significant change was observed in the WPI group. This indirectly suggests that whey protein may exert a superior therapeutic effect against obesity relative to soy protein. To investigate the underlying molecular mechanisms, we performed additional studies. Energy intake, changes in appetite-related hormone levels, lipid excretion, and adipose tissue alterations are well-established factors directly linked to the onset and progression of obesity. In this study, relevant parameters were measured. Our results showed no significant differences in food and energy intake between the WPI and SPI groups. However, it should be noted that individual mouse food and energy intake values were derived from cage-level measurements. This may introduce potential measurement errors; therefore, these findings warrant cautious interpretation. Leptin, adiponectin, CCK, GLP-1, GIP, and ghrelin are key appetite-regulating hormones. Ghrelin is an orexigenic hormone that stimulates food intake when elevated. Conversely, leptin, adiponectin, CCK, GLP-1, and GIP function as anorexigenic hormones, by increased concentrations suppressing food intake [49]. In the present study, SPI-fed mice exhibited significantly lower serum leptin concentrations and higher GIP concentrations relative to the WPI group. Notably, elevated circulating GIP concentrations have been reported in individuals with obesity compared with healthy controls [50]. GIP promotes obesity pathogenesis through the following mechanisms: (1) enhancing postprandial energy storage via nutrient partitioning; (2) increasing lipoprotein lipase activity and regulating its release in adipose tissue through receptor binding, stimulating de novo lipogenesis and lipid storage; (3) facilitating free fatty acid re-esterification; and (4) enhancing intestinal nutrient absorption [51,52,53]. Consequently, the intergroup disparity in serum GIP concentrations likely mediates, at least in part, the divergent anti-obesity potency of WPI versus SPI. Analysis of fecal lipids (TG and TC) revealed significantly elevated TG levels in the WPI group compared with the SPI group. This observation suggests that whey protein, relative to soy protein, may confer superior anti-obesity efficacy, potentially mediated by enhanced fecal TG excretion. Notably, fecal TC levels were significantly lower in the WPI group compared with the SPI group, demonstrating an inverse trend relative to TG. Previous studies indicate that soy protein supplementation effectively reduces circulating cholesterol levels [54]. Compared with total dairy protein, soy protein demonstrates superior cholesterol-lowering efficacy [55]. This effect may be mediated by enhanced fecal cholesterol excretion [56,57]. Furthermore, relative to the WPI group, increased weight of BAT and iWAT, enhanced lipid droplet accumulation in BAT, and significantly enlarged adipocyte areas in eWAT were observed in the SPI group. These findings provide additional evidence for the superior anti-obesity effects of whey protein over soy protein, although the molecular mechanisms underlying this difference require further investigation.

There are several limitations in the present study. Firstly, we did not measure fasting blood glucose and insulin sensitivity after the 16-week HFD feeding (used to establish the obesity model) because required fasting procedure could cause significant body weight fluctuations in mice. However, significantly elevated fasting blood glucose and HOMA-IR levels were observed in both HFD and WPI groups compared to the control group after 6 weeks of dietary intervention. These findings suggest that the 16-week HFD feeding successfully induced IR. Secondly, the duration of the HPDs intervention was relatively short, which may limit the interpretation of its long-term effects. Thirdly, the OPLS-DA model is at risk of overfitting, which may compromise the reliability of the inferred global metabolic separation results.

## 5. Conclusions

In summary, our findings demonstrated that soy protein outperformed whey protein in ameliorating IR in HFD-induced obese mice, potentially mediated through modulation of the PI3K-GLUT4 pathway and glycerophospholipid metabolism. Moreover, soy protein and whey protein exhibited comparable anti-obesity efficacy.

## Data Availability

The original contributions presented in the study are included in the article; further inquiries can be directed at the corresponding authors.

## Supplementary Materials

The following supporting information can be downloaded at: https://www.mdpi.com/article/10.3390/nu17213427/s1, Table S1: Compositions of experimental diets; Table S2: Differentially abundant metabolites in serum between the WPI and SPI groups; Table S3: Differentially abundant metabolites in liver between the WPI and SPI groups; Figure S1: PCA score plots of serum metabolic profiles (A) and hepatic metabolic profiles (B) among WPI and SPI groups. *n* = 6 per group.
