# Supplementary material for: Soy Protein Outperforms Whey Protein in Ameliorating Insulin Resistance but Not Obesity in High-Fat Diet-Induced Obese Mice
Source: Nutrients. 2025 Oct 31;17(21):3427. doi: 10.3390/nu17213427 (PMC12608677; doi:10.3390/nu17213427)
Supplement: Supplementary file 1 [file nutrients-17-03427-s001.zip › nutrients-3929877-supplementary.pdf]

Supplementary Table S1 Compositions of experimental diets

	Control	HFD	WPI	SPI
**Ingredient (g)**				
Casein	200	200	0	0
Whey protein isolate	0	0	200	0
Soy protein isolate	0	0	0	200
L-Cystine	3	3	3	3
Corn starch	506.2	0	0	0
Maltodextrin 10	125	125	125	125
Sucrose	72.8	72.8	72.8	72.8
Cellulose, BW200	50	50	50	50
Soybean oil	25	25	25	25
Lard	20	245	245	245
Mineral mix S10026B	50	50	50	50
Vitamin mix V10001C	1	1	1	1
Choline bitartrate	2	2	2	2
FD&C yellow dye #5	0.04	0	0	0
FD&C blue dye #1	0.01	0.05	0.05	0.05
Total (g)	1055.05	773.85	773.85	773.85
**Energy (kcal %)**				
Protein	20%	20%	20%	20%
Carbohydrate	70%	20%	20%	20%
Fat	10%	60%	60%	60%
Energy density (kcal/g)	3.85	5.22	5.22	5.22
Total (kcal)	4037	4037	4037	4037

WPI, whey protein isolate; SPI, soy protein isolate.

Supplementary Table S2 Differentially abundant metabolites in serum between the WPI and SPI groups

Metabolite	Formula	SPI/WPI	Trend	*P*-value
Phosphocholine	$C_5H_{15}NO_4P^+$	1.02±0.01	Up	0.004

The peak height of each metabolite was $\log_{10}$-transformed and normalized to calculate the fold change of differentially abundant metabolites between the WPI and SPI groups. WPI, whey protein isolate; SPI, soy protein isolate. Data are represented as mean ± SEM (n = 6).

Supplementary Table S3 Differentially abundant metabolites in liver between the WPI and SPI groups

Metabolite	Formula	SPI/WPI	Trend	*P*-value
Chenodeoxycholic acid	$C_{24}H_{40}O_4$	0.54±0.01	Down	< 0.001
hyodeoxycholic acid	$C_{24}H_{40}O_4$	0.86±0.03	Down	0.002
Decanoyl-L-carnitine	$C_{17}H_{33}NO_4$	0.82±0.04	Down	0.023

Leucylproline	$C_{11}H_{20}N_2O_3$	0.92±0.01	Down	0.046
D-Glucosaminic acid	$C_6H_{13}NO_6$	0.88±0.03	Down	0.045
Phosphocholine	$C_5H_{15}NO_4P^+$	1.08±0.03	Up	0.048
Cholic acid	$C_{24}H_{40}O_5$	0.88±0.04	Down	0.037

The peak height of each metabolite was $\log_{10}$-transformed and normalized to calculate the fold change of differentially abundant metabolites between the WPI and SPI groups. WPI, whey protein isolate; SPI, soy protein isolate. Data are represented as mean ± SEM (n = 6).

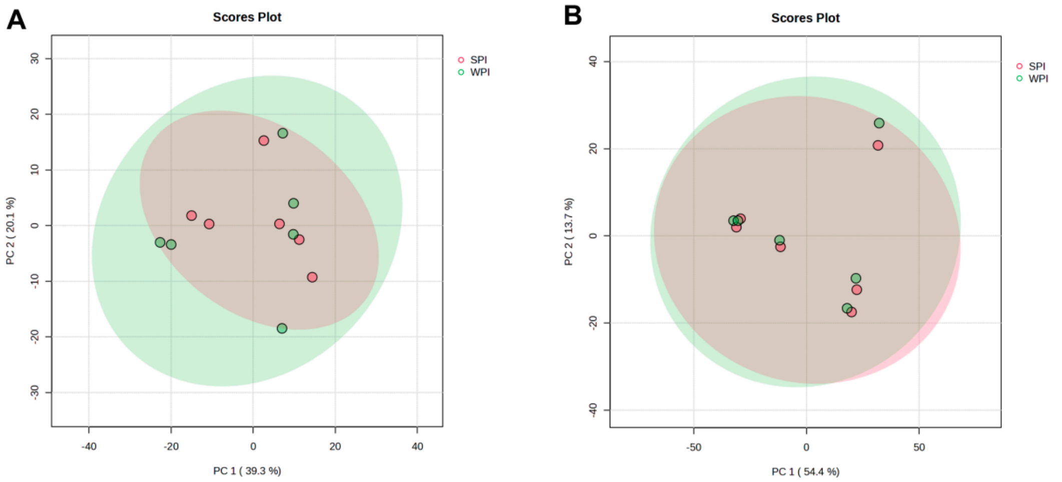

Supplementary Figure S1 PCA score plots of serum metabolic profiles (A) and hepatic metabolic profiles (B) among WPI and SPI groups. n = 6 per group.
